# Aging and Decision Making: New Insights Regarding Information Evaluation and Management

**DOI:** 10.1093/geroni/igaf122.1398

**Published:** 2025-12-31

**Authors:** Julia Nolte, Corinna Löckenhoff, JoNell Strough

## Abstract

Age-related cognitive shifts in information processing and memory capacities are associated with more frugal information management: Older adults engage with some rather than all available information before making a decision. At the same time, motivational shifts prompt older adults to selectively engage with information that is personally meaningful or emotionally salient. The individual talks in this symposium document how these phenomena manifest themselves across a range of decision-related contexts. With respect to personal meaningfulness, Nolte, Shavit, and Ng demonstrate that older adults express a stronger preference for information or options that reaffirm personally held beliefs than is the case for younger age groups. Wild, Lachs, and Löckenhoff show that by comparison, older adults are more likely to prepare for healthcare decisions by developing written agendas of concerns and questions that are personally meaningful to them. With respect to emotional salience, Liao, Lu, Ma, Guo, Zhou, Sun, Li, and Zhang document that older adults process age-related stereotypes differently depending on valence: When exposed to positive rather than negative age-related stereotypes, older adults expect to encounter lower levels of risk within financial decisions. By contrast, Chen, Toh, Shavit, and Carstensen observe that older adults’ financial decisions are susceptible to negatively valenced information: Compared to middle-aged adults, older adults make larger donations when faced with the negative consequences of their failure to donate (versus the positive consequences of donating). To integrate insights across these studies and identify directions for future research, Strough will contextualize the presentations against the extant aging and decision-making literature. Judgement and Decision Making Interest Group Sponsored Symposium

